# The analgesic mechanism of electroacupuncture at the central level for neuropathic pain: a review of studies based on animal experiments

**DOI:** 10.3389/fneur.2025.1587471

**Published:** 2025-05-29

**Authors:** Pengfei Qi, Quan Li, Mingyuan Han, Yang Cui, Xinyu Zhou, Zhongren Sun, Shuo Ding, Mengdi Yu, Hongbo Zhang, Hongna Yin

**Affiliations:** ^1^Heilongjiang University of Chinese Medicine, Harbin, China; ^2^The Second Affiliated Hospital of Heilongjiang University of Chinese Medicine, Harbin, China

**Keywords:** electroacupuncture, neuropathic pain, central mechanisms, spinal dorsal horn, brain regions, animal models

## Abstract

This article analyzes the progress of animal experiments on the analgesic mechanism of electroacupuncture (EA) at the central level for neuropathic pain (NP) in the past 10 years, and summarizes the analgesic mechanism of EA at the central level for NP. EA, as a safe and reliable treatment, can treat NP by regulating the release of nociceptive neurotransmitters and receptors, upregulating the expression of non-coding RNA (ncRNA), inhibiting the activation of microglia, Ca^2+^/calmodulin kinase II (CaMKII) phosphorylation, dendritic spine remodeling, endoplasmic reticulum stress (ERS), and glucose metabolism. NP is a type of pain caused by various diseases. Pain caused by stroke, spinal cord injury, postherpetic neuralgia (PHN), diabetes, and chemotherapy-induced neuropathy all fall into the category of NP, which makes the treatment of NP very challenging. At present, EA research on the treatment of NP is more focused on the mechanism of the dorsal horn of the spinal cord, and there are relatively few animal experiments at the level of the central brain region. There is also a lack of clinical trials using human subjects and relevant biochemical indicators. In the future, electrophysiology, neuron tracing, and multi-omics techniques combined with emerging technologies such as artificial intelligence should be used to further improve the analgesic mechanism of EA on the central level for NP, making EA the best treatment for NP.

## 1 Introduction

Pain is a sensory and emotional experience associated with, or described by, tissue damage (1). The International Association for the Study of Pain (IASP) defines pain as an unpleasant sensory and emotional experience associated with actual or potential tissue damage, or with the description of such damage. Neuropathic pain (NP) is defined as pain arising from lesions or diseases of the somatic sensory nervous system (2, 3). NP refers to pain caused by secondary damage or dysfunction, and its pathogenesis involves the peripheral nervous system and the entire central nervous system (CNS), including the spinal cord and brain areas (4). Pain caused by stroke, spinal cord injury, postherpetic neuralgia (PHN), diabetes, and chemotherapy-induced neuropathy all fall into the category of NP (5, 6). A survey conducted recently indicates that (7), approximately 10% of the global population has suffered from NP at some point in their lives. This not only seriously affects the quality of life of those affected, but also places a significant burden on the global economy. Current research into the mechanisms of NP is mainly based on animal experiments. Common disease models include the spinal nerve ligation (SNL) model, the selective nerve injury (SNI) model, and the chronic compression injury (CCI) model of the sciatic nerve (8). In recent years, with the deepening of research on the peripheral nervous mechanism of NP and the increasing maturity of neuromodulation techniques such as transcranial magnetic stimulation and transcranial direct current stimulation, the research on NP is not limited to peripheral nervous mechanisms alone. There is an increasing amount of research on central nervous mechanisms, which has gradually become a hotspot in the field of research on the mechanism of NP (9). NP is a chronic and persistent symptom that has long been a major challenge in the field of global medicine due to its high incidence and low treatment rate. Currently, the first-line treatment drugs for NP are mainly antiepileptic drugs and antidepressants. They can also be used in combination with opioids to treat NP. Although these methods can effectively relieve NP, long-term use may cause drug tolerance and dependence, as well as adverse reactions such as nausea, vomiting, and constipation (10, 11). Therefore, the search for safer and more effective treatment strategies is crucial for the treatment of NP. In recent years, with the deepening of research on traditional Chinese medicine, a large number of literature studies have reported that EA has significant curative effects in the treatment of NP without adverse reactions (12–14). However, acupuncture analgesia is a complex network regulation mechanism from the peripheral to the central nervous system (CNS), involving the entire nervous system. Many biologically active substances are involved in the regulation of EA on NP. At present, the analgesic mechanism of EA on NP has not been clearly established, so it is necessary to summarize the mechanism of EA in the treatment of NP.

This article summarizes the central mechanism of EA in the treatment of NP by reviewing relevant literature from PubMed, Web of science, and the China Knowledge Infrastructure project. The aim is to clarify the central mechanism of EA in the treatment of NP and provide a basis for further exploration of EA in the treatment of NP. The following will systematically explain the central mechanism of EA for NP pain relief, focusing on the release of neurotransmitters and receptors related to pain perception, activation of microglia, Ca^2+^/calmodulin kinase II (CaMKII) phosphorylation, non-coding RNA (ncRNA) expression, dendritic spine remodeling, endoplasmic reticulum stress (ERS), and glucose metabolism.

## 2 The central mechanism of NP

**Figure 1:**
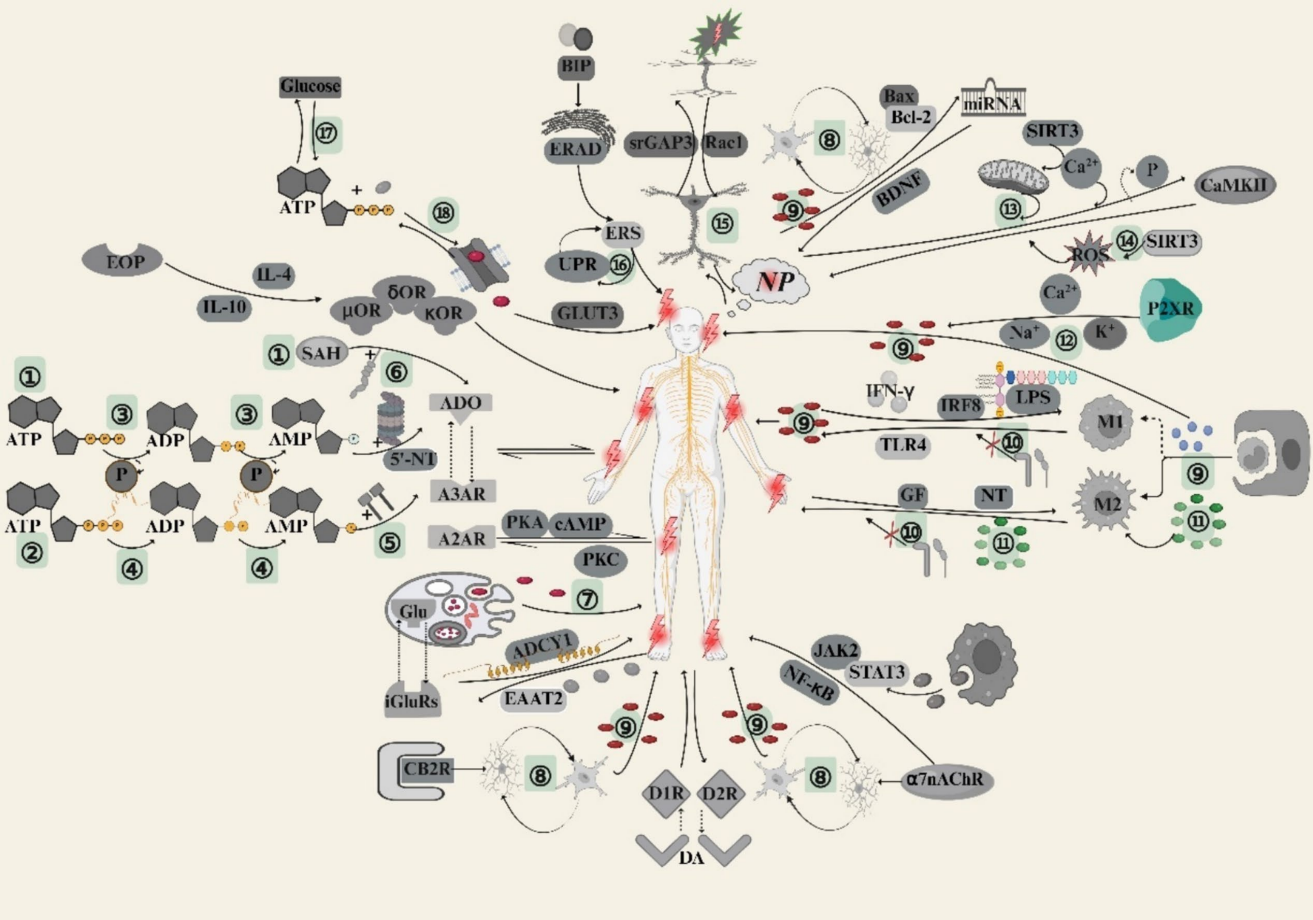
Key mechanisms involved in the onset and progression of NP. The figure was drawn using biorender (https://www.biorender.com/). ①: Intracellular pathway formed by ADO; ②: Extracellular pathway formed by ADO; ③: Dephosphorylation process; ④: CD39 hydrolysis; ⑤: CD73 hydrolysis; ⑥: SAH hydrolase hydrolysis; ⑦: Syt-1 controls neurotransmitter exocytosis; ⑧: Activation of microglia; ⑨: Release of pro-inflammatory factors; ⑩: PD-L1 and PD1 are absent; ⑪: Anti-inflammatory factor release; ⑫: Activation of ion channels in the cell membrane (influx of Na^+^ and Ca^2+^ and efflux of K^+^); ⑬: Regulates lysine acetylation in mitochondria; ⑭: SIRT3 is carbonylated or inactivated; ⑮: Dendritic spine remodeling; ⑯: The conformational binding state of BIP with PERK, IRE1 and ATF6 is separated; ⑰: Glucose metabolism; ⑱: Neuronal signal transmission (generation of action potentials and synaptic transmission, etc.).

The central mechanisms of NP occurrence and progression mainly involve the release of neurotransmitters and activation of receptors related to pain perception, activation of microglia, CaMKII phosphorylation, ncRNA expression, dendritic spine remodeling, ERS, and glucose metabolism. The main central mechanisms of NP are shown in Figure 1.

### 2.1 Regulation of the release of neurotransmitters and activation of receptors related to pain perception

NP perception, transmission, and processing involve numerous pain-related neurotransmitters, including endogenous opioids (EOP), adenosine (ADO), and glutamate (Glu). In addition, the release of EOP receptors, ADO receptors, cannabinoid receptors (CBR), dopamine receptors (DR), and A7 nicotinic acetylcholine receptors (α7nAChR) also has an analgesic effect on NP (15–19).

#### 2.1.1 Regulation of EOP and its receptor expression

EOP, which is composed of *β*-endorphin (β-EP), enkephalin and dynorphin, acts as an important regulator of opioid receptor expression and activity in the central nervous system. It is involved in the analgesic effects of NP by binding to *μ*, *δ* and *κ* opioid receptors (μOR, δOR and κOR) (20). Research shows that (21, 22), EOP is essential for maintaining normal levels and activity of opioid receptors. Activating the expression of *β*-EP, enkephalin, and dynorphin and their receptors can all produce a good analgesic effect on NP. In CCI model rats, intrathecal injection of the μOR agonist DPDPE can effectively relieve NP, and intrathecal injection of the μOR antagonist BNTX reverses the analgesic effect of DPDPE (23). Intrathecal injection of transforming growth factor-*β* (TGF-β) effectively alleviates NP in SNI model mice by upregulating the expression of β-EP on the presynaptic membrane, enkephalin, and μOR and δOR on the postsynaptic membrane (24). Beta-endorphin-producing neurons in the arcuate nucleus of the hypothalamus (ARC) are involved in the regulation of NP by synthesizing and releasing beta-endorphin. In rats with a trigeminal neuralgia (CCI-ION) model, the synthesis of beta-endorphin by beta-endorphin-producing neurons in the ARC is reduced, and specific activation of beta-endorphin in the ARC has a good analgesic effect on NP (25). In SNL model rats, intrathecal injection of the GPR40 agonist GW9508 can effectively increase the mechanical withdrawal threshold (MWT) and thermal withdrawal latency (TWL) of the ipsilateral hind paw in a dose-dependent manner, and can stimulate microglia in the dorsal horn of the spinal cord to express IL-10 and *β*-EP, thereby effectively alleviating NP (26). In addition, the anti-inflammatory cytokines IL-4 and IL-10 can relieve NP by activating the release of EOP and its related receptors. The anti-inflammatory cytokine IL-4 effectively relieves NP by inducing M2 macrophages to continuously produce EOP (27). IL-10 in microglia effectively alleviates NP in rats with SNL model by inhibiting excitatory synaptic transmission at pre- and postsynaptic muOR in the dorsal horn of the spinal cord (28). This indicates that activation of the IL-10/*β*-EP signaling pathway can produce an analgesic effect on NP.

#### 2.1.2 Up-regulation of the expression of ADO and its receptors

As a neurotransmitter, ADO is present both inside and outside the cells of the body. Inside the cell, ADO is one of the end products formed after the degradation of adenosine triphosphate (ATP). The phosphorylation of ATP forms adenosine diphosphate (ADP), which continues to be phosphorylated to form adenosine monophosphate (AMP). AMP is finally formed into ADO by the action of 5′-nucleotidase. Meanwhile, adenosine-L-homocysteine (SAH) can also be hydrolyzed by intracellular SAH hydrolase to form ADO (29, 30). Extracellularly, the extracellular nucleoside triphosphate hydrolase (CD39) converts ATP and ADP to AMP, which is then hydrolyzed by the extracellular-5′-nucleotidase (CD73) to form ADO (31). ADO is involved in the analgesic process of NP by binding to its four highly related G protein-coupled receptors [adenosine A1 receptors, adenosine A2A receptors (A2ARs), adenosine A2B receptors, and adenosine A3 receptors (A3Rs)] (32, 33). Among them, A2AR, which is widely distributed in the CNS, is mainly involved in the central analgesic mechanism of NP (34). In rats with a model of spinal nerve root avulsion pain, a single intrathecal injection of the A2AR agonists CGS21680 and ATL313 can effectively increase MWT and reverse MWT for at least 6 weeks (35). In CCI model rats, a single intrathecal injection of A2AR agonists ATL313 or CGS21680 can also reverse MWT for at least 4 weeks. Intrathecal injection of ATL313 can also have an analgesic effect on NP by activating the protein kinase A (PKA)/protein kinase C (PKC) signaling pathway, and after intrathecal injection of PKA and PKC inhibitors, reversed the analgesic effect of the A2AR agonist ATL313 on NP (36). Therefore, activating A2AR can not only directly relieve NP, but also produce an analgesic effect on NP by activating the PKA/PKC signaling pathway. In addition, after A2AR is activated, it can also increase the intracellular concentration of cAMP by accelerating the formation of the intracellular second messenger cyclic adenosine monophosphate (cAMP). As the intracellular concentration of cAMP increases, it further activates PKA to release neurotransmitters to relieve NP (37, 38).

A3R, which is widely distributed in the peripheral nervous system, is also effective in relieving pain after activation, even though its expression in the CNS is relatively low. It has a similar analgesic effect on NP induced by SNI, CCI and chemotherapeutic drugs (39–41). In rats with a sciatic nerve injury model, intraperitoneal injection of the A3AR agonist IB-MECA effectively alleviates NP by increasing MWT and inhibiting activation of microglia in the dorsal horn of the spinal cord (42). In SNI model mice, intrathecal injection of IB-MECA alleviates NP by significantly increasing MWT, TWL, and the expression level of ADO in the dorsal horn of the spinal cord. Intrathecal injection of the A3AR antagonist MRS1523 reverses this phenomenon (43). This indicates that accelerating the binding of ADO and A3R in the dorsal horn of the spinal cord can have a positive analgesic effect on NP.

#### 2.1.3 Modulation of the expression of Glu and its receptors

Glu is a common excitatory neurotransmitter in the nervous system of mammals. In the brain of mammals, Glu accounts for about 50–80% of all neurotransmitters. Glu is widely involved in the transmission of excitatory signals between synapses by binding to ionotropic glutamate receptors (iGluRs) and metabotropic glutamate receptors in presynaptic and postsynaptic neurons (44, 45). iGluRs include four types of receptors: *α*-amino-3-hydroxy-5-methyl-4-isoxazolepropionic acid receptors, the kainate receptors, the N-methyl-D-aspartate receptor (NMDAR) and the orphan glutamate *δ* receptor. After binding to iGluRs, Glu mainly mediates the transmission of pain signals as an ion channel. The NMDAR is widely distributed in the brain and spinal cord, and inhibiting the expression of NMDAR can effectively relieve NP (46). In SNL model rats, intrathecal injection of the selective NMDAR antagonist Ro25-6981 alleviates NP (47). In a cisplatin-induced neuropathic pain (CINP) mouse model, NMDAR subtype GluN2D knockout mice have reduced sensitivity to NP. Injection of GluN2D inhibitors UBP141 and UBP1700 can effectively relieve NP by increasing MWT (48). At the same time, glutamate transport protein-2 (EAAT-2), which is located in neurons and astrocytes, can inhibit the expression of iGluRs, thereby inhibiting the transmission of excitatory signals and producing analgesic effects on NP (49). In addition, NP can be alleviated by regulating the expression level of downstream proteins of NMDAR in the CNS. As a downstream protein of NMDAR, downregulating the expression level of ADCY1, a member of the adenylate cyclase (ADCY) family, can effectively alleviate NP (50, 51). Modulating the release of glutamate also has a therapeutic effect on NP. Synaptic vesicle protein 1 (Syt-1), as a synaptic vesicle protein regulating neurotransmitter exocytosis, can effectively alleviate NP by downregulating Glu expression (52, 53). However, when NP occurs, the expression of Glu in different brain regions is not always consistent, which indirectly leads to the complexity of the central brain region mechanism of NP. In SNI model mice and CCI model rats, the expression level of Glu in the anterior cingulate cortex (ACC) is elevated. Inhibiting the expression of Glu in the ACC can have an analgesic effect on NP (54, 55). In contrast, SNI model mice have significantly lower Glu levels in the ventrolateral periaqueductal gray (vlPAG) of the midbrain. Upregulating Glu levels in the vlPAG can effectively alleviate NP (56).

#### 2.1.4 Upregulation of the expression of CBR

Cannabinoids, a neurotransmitter derived from the cannabis plant, can relieve NP by regulating endogenous ligands in the endocannabinoid system (ECS), cannabinoid receptors, and enzymes responsible for cannabinoid synthesis and degradation (57, 58). Cannabis extract relieves NP by upregulating the expression level of cannabinoid receptor 2 (CB2R) in the hippocampus and cerebral cortex of rats (59). In addition to CB2R, there is another important receptor in the ECS, the cannabinoid receptor 1 (CB1R). Both belong to the family of G protein-coupled receptors and upregulating the expression of CB1R can also relieve NP. However, long-term use can cause symptoms of cannabinoid-like CNS psychosis, which greatly limits the use of CB1R agonists in NP. CB2R relieves NP by inhibiting the release of pro-inflammatory factors from microglia, and there are no significant adverse reactions (60, 61). Therefore, CB2R is often used as an effective target for the treatment of NP. Research shows (62–64), Intraperitoneal injection of CB2R agonists LY2828360 and AM1710 can inhibit NP caused by the chemotherapeutic drug paclitaxel (PTX). In SNI model rats, continuous intrathecal injection of the CB2R agonist PM226 effectively relieves NP by promoting the transformation of microglia in the dorsal horn of the spinal cord from an inflammatory to an anti-inflammatory stage (65). Meanwhile, intrathecal injection of the CB2R agonist AM1710 alleviates NP by reducing the expression of IL-1β receptors in the dorsal horn of the spinal cord of CCI model mice, while intraperitoneal injection of the CB2R antagonist AM630 aggravates NP (66, 67). This shows that upregulating the expression level of CB2R can effectively alleviate NP, while inhibiting the expression level of CB2R can aggravate NP.

#### 2.1.5 Modulation of the expression of DR

Dopamine (DA) is one of the most important catecholamine neurotransmitters synthesized by cells in the ventral midbrain of the CNS (68). Inhibiting DA neurons can aggravate NP caused by Parkinson’s disease (69). After being released, DA will mediate the occurrence and progression of NP by binding to its dopamine type 1 receptor (D1R) and dopamine type 2 receptor (D2R). Low concentrations of DA binding to D1R aggravate NP, but high concentrations of DA binding to D2R can effectively alleviate NP (70). In the CCI rat model, a single intrathecal injection of the D1R antagonist SCH-23390 and the D2R agonist quinpirole can both relieve NP (71, 72). In CCI-ION model mice, inhibiting the expression of D1R and enhancing the expression of D2R in the ACC brain region can effectively alleviate the NP of CCI-ION mice (73).

#### 2.1.6 Upregulation of α7nAChR expression

The nicotinic acetylcholine receptor (nAChR) is an acetylcholine (ACh) receptor composed of 17 different subunits. These subunits combine to produce subtypes with different pharmacological properties (74). Among them, α7nAChR is the most highly expressed nAChR subtype in mammals, and increasing the expression level of α7nAChR can effectively relieve NP. In CCI model rats, long-term oral administration of the α7nAChR agonist PNU-282987 can relieve NP through the mechanism of nAChR antagonist inhibition (75). As a core member of the cholinergic anti-inflammatory pathway, ACh released into the bloodstream in response to vagal nerve stimulation also participates in the regulation of the cholinergic anti-inflammatory pathway by binding to the *α*7nAChR expressed on macrophages (76). The *α*7nAChR, which is widely distributed in the CNS, also relieves NP by reducing the production and release of inflammatory cytokines (IL-1β, IL-6, IL-18) and tumor necrosis factor-α (TNF-α) (77). The α7nAChR expressed in response to stimulation by macrophages is also involved in the anti-inflammatory response and thus in the analgesic effect on NP by activating the janus-kinase-2 (JAK-2)-signal transducer and activator of transcription-3 (STAT3) pathway (JAK2-STAT3) and inhibiting nuclear factor κB (NF-κB) (78). The persistent inflammatory response can also mediate NP via activated microglia (79). In CCI model rats, activation of α7nAChR in spinal dorsal horn microglia can effectively alleviate NP by reducing the release of pro-inflammatory cytokines and upregulating the expression of anti-inflammatory cytokines (80).

### 2.2 Inhibition of activation of microglia

Microglia cells mainly mediate NP by releasing pro-inflammatory factors and regulating the expression of the ionotropic purinergic receptor (P2XR). Inhibiting the release of pro-inflammatory factors by microglia cells and down-regulating the expression of P2XR can effectively relieve NP.

#### 2.2.1 Inhibition of the release of pro-inflammatory factors by microglia

The CNS is mainly composed of neurons and glial cells. In the CNS, microglial cells, astrocytes, and oligodendrocytes together form a large number of glial cells (81). Research shows that (82), Microglia cells derived from the yolk sac act as immune cells in the CNS. They not only have anti-inflammatory and pro-inflammatory abilities, but also phagocytic effects. Microglia cells are the first glial cells in the central system to respond and play a vital role in mediating NP. Microglia can have both a promoting and inhibiting effect on NP through phenotypic transformation. Under normal circumstances, M1 and M2 microglia are in a state of dynamic equilibrium. When M1 and M2 microglia are activated, the original equilibrium is disrupted, which can trigger NP (83). M1 microglia, as participants in nociception, are induced by substances such as interferon (IFN-*γ*) and lipopolysaccharide (LPS), and increase the excitability of neurons by releasing pro-inflammatory cytokines or other nociceptive mediators, thereby aggravating NP. In contrast, M2 microglia, induced by anti-inflammatory factors, effectively relieve NP by releasing anti-inflammatory mediators, cell growth factors and neurotrophic factors (84, 85). After nerve damage, interferon regulatory factor 8 (IRF8) upregulates the number of M1 microglia in the spinal cord. The activated M1 microglia aggravates NP by triggering an inflammatory response (86, 87). The chemotherapeutic drug PTX exacerbates NP by activating Toll-like receptor 4 (TLR4) in the spinal cord and its downstream NF-κB signaling pathway, which accelerates the release of pro-inflammatory cytokines (88, 89). Brain-derived neurotrophic factor (BDNF) is a basic neurotrophic factor that is widely involved in signal transduction in microglia in the dorsal horn of the spinal cord. Nociceptive stimuli further activate microglia type M1 by upregulating BDNF expression in the dorsal horn of the spinal cord, reducing the anti-inflammatory function of microglia and enhancing excitability after nociceptive stimuli, aggravating NP (90). In addition, the absence of programmed death ligand 1 (PD-L1) and programmed death receptor 1 (PD-1) can promote the polarization of M1 microglia and thus aggravate NP, while increasing the expression levels of PD-L1 and PD-1 can effectively alleviate NP by promoting the polarization of M2 microglia (91).

#### 2.2.2 Suppression of P2XR expression in microglia

P2 purinergic receptors are important cell signaling factors that are divided into ionotropic P2X receptors and metabotropic P2Y receptors. Ionotropic P2X receptors include seven subtypes, including P2X1R-P2X7R (92). Ionotropic P2X receptors are found in almost all tissues and organs of the body. About 70% of P2X7R are expressed in the CNS, such as the spinal cord and brain. After being activated by extracellular ATP, P2XR mediates the influx of Na^+^ and Ca^2+^ and the efflux of K^+^, thereby activating ion channels in the cell membrane and participating in the transmission of pain signals (93). Research shows (94), P2XR activation induces NP by opening ion channels in the cell membrane and activating microglia. Upregulating P2X4R expression in microglia in the dorsal horn of the spinal cord can exacerbate NP (95). In rats with a streptozotocin (STZ)-induced diabetic neuropathic pain (DNP) model, intrathecal injection of the P2X4R antagonist 5-BDBD relieved NP by reducing the expression levels of BDNF, IL-1β and TNF-*α* in the dorsal horn of the spinal cord (96). In addition, activation of microglia in the dorsal horn of the spinal cord can cause abnormal neuronal activity by increasing the expression level of P2X7R and further releasing inflammatory factors such as IL-18, IL-1β, and TNF-*α*, which enhance the transmission of harmful neurons at the synapses and aggravate NP (97). Intrathecal injection of the selective P2X7R antagonist A438079 reduces the expression levels of IL-1β and IL-18 in the dorsal horn of the spinal cord by inhibiting the activation of microglia in CCI model rats, thereby alleviating NP (98).

### 2.3 Inhibition of CaMKII phosphorylation

CaMKII is a multifunctional serine/threonine protein kinase. The earliest research on CaMKII focused on enhancing synaptic plasticity in the hippocampus and its involvement in learning and memory (99, 100). Recent studies have found that CaMKIIα, as one of the main subtypes of the CaMKII family, is widely distributed in the dorsal horn of the spinal cord and various brain regions. Both the phosphorylation of CaMKIIα itself and its activation after Ca^2+^ influx can aggravate NP (101). In CCI model rats, intrathecal injection of the CaMKII-specific inhibitor m-AIP can downregulate hippocampal CaMKIIα and pCaMKIIα protein expression levels, thereby alleviating NP (102). In addition, the phosphorylation of CaMKIIα is regulated by Sirtuin 3 (SIRT3). SIRT3 is a mitochondrial enzyme that protects against NP by regulating mitochondrial lysine acetylation mediated by NP. Maintaining SIRT3 activity is essential for alleviating NP (103). A transcriptome sequencing of the spinal cord tissue of DNP mice revealed that SIRT3 may be a key molecule mediating NP, and that inhibiting the activation of microglia in the dorsal horn of the spinal cord of DNP model rats and the protein level of SIRT3 can aggravate NP (104). In DNP model rats, the expression levels of MWT, TWL and SIRT3 in the dorsal horn of the spinal cord are significantly reduced. Intrathecal injection of LV-SIRT3 can effectively alleviate NP by increasing MWT and TWL and upregulating SIRT3 expression in the dorsal horn of the spinal cord in DNP model rats (105). Spinal cord SIRT3 carbonylation or inactivation can also further activate pCaMKII by increasing the level of reactive oxygen species (ROS), aggravating NP (106).

### 2.4 Upregulation of the expression of microRNAs in ncRNAs

ncRNA is a type of RNA that does not have the ability to be translated into protein (107). ncRNAs are divided into two categories based on whether their sequence length exceeds 200 nt: long-chain and short-chain. Long-chain ncRNAs are RNAs with a length exceeding 200 nucleotides, while RNAs with a length less than 200 nucleotides are called short-chain ncRNAs (108). Short-chain ncRNAs are mainly composed of microRNAs (miRNAs), ribosomal RNAs, small nuclear RNAs, and piwi-interacting RNAs. Among them, miRNAs are involved in and mediate the occurrence and progression of NP through various mechanisms, such as immune cell infiltration, neuroinflammatory response, and ion channel expression (109, 110). MiR-206 is a 21-nucleotide miRNA with neuromodulatory functions. It has two mature isoforms, miR-206-3p and miR-206-5p (111, 112). In CCI model rats, miR-206 can alleviate NP by reducing the expression level of BDNF and the content of pro-inflammatory cytokines TNF-*α*, IL-1β, and IL-6 (113). Therefore, increasing the expression level of miR-206 can effectively alleviate NP. miR-124, which also acts as an anti-inflammatory regulator, can alleviate NP by inhibiting microglial activation, while miR-155 can aggravate NP by activating microglia to release pro-inflammatory factors (114). Inhibiting the expression of miR-155 can alleviate NP by inducing the transformation of the M1 phenotype of microglia to the M2 phenotype. In the SNL model rat, intrathecal injection of a miR-155 inhibitor can alleviate NP by increasing the MWT, accelerating the activation of M2 microglia, reducing the expression levels of IL-1β and TNF-*α*, and inhibiting the activation of M1 microglia (115). In addition, intrathecal injection of miR-124-3p inhibitors can aggravate NP by promoting the mRNA and protein expression levels of early growth response gene 1 (EGR1) in the dorsal horn of the spinal cord (116).

### 2.5 Inhibition of dendritic spine remodeling

Dendritic spines are small, dynamically structured protrusions located on the dendrites of neurons. They not only contain a dense cytoskeleton, but also transmembrane and scaffold molecules (117). During continuous neuronal firing, the shape (filamentous, slender, stubby and mushroom-shaped), distribution and density of dendritic spines are constantly changing. This dynamic process is known as dendritic spine remodeling (118–120). SLIT-ROBO Rho GTPase Activating Protein 3 (SRGAP3) and Rho family member Ras-related C3 botulinum toxin substrate 1 (Rac1) are involved in mediating dendritic spine remodeling by regulating cytoskeleton elements-actin (121). SrGAP3 and Rac1 play complementary roles at different stages of the dendritic spine life cycle and are jointly involved in dendritic spine remodeling. SrGAP3 mainly mediates the formation of dendritic spines and determines the maturation of dendrites, while Rac1 is mainly involved in the maintenance of mature dendritic spines (122). Research shows (123, 124), Reducing Rac1 activity can effectively inhibit dendritic spine remodeling and reduce neuronal hyperexcitability by disrupting dendritic spine morphology and alleviating NP. In CCI and SCI model rats, intrathecal injection of Rac1 selective inhibitor NSC23766 can effectively alleviate NP by improving MWT, reducing Rac1 expression levels in the dorsal horn of the spinal cord, and the density and morphology of dendritic spines, thereby inhibiting neuronal hyperexcitability and dendritic spine remodeling (125, 126). At the same time, In DNP model rats, intrathecal injection of Rac1 selective inhibitor NSC23766 effectively alleviates NP by increasing MWT, reducing the number of mushroom-shaped dendritic spines in the dorsal horn of the spinal cord that promote signal transmission, inhibiting neuronal excitability, and dendritic spine remodeling (127). In addition, srGAP3 is involved in dendritic spine remodeling by regulating Rac1 activity, thereby mediating NP (128). Upregulating srGAP3 expression can promote the formation of immature dendritic spines, inhibit Rac1 activity, and effectively alleviate NP (129, 130).

### 2.6 Inhibition of ERS

The endoplasmic reticulum (ER), an important organelle for protein synthesis and calcium storage in eukaryotic cells, mediates the protein quality control system by removing misfolded proteins through the endoplasmic reticulum-related degradation pathway and further degrading them, thereby maintaining protein homeostasis (131). However, when persistent misfolded proteins cannot be transported to the cytoplasmic proteasome system for degradation in time, they gradually accumulate in the ER lumen, which can lead to ERS. Persistent ERS triggers the unfolded protein response (UPR) under the action of protein kinase R-like endoplasmic reticulum kinase (PERK), inositol requiring enzyme 1 (IRE1), and activating transcription factor 6 (ATF-6) to restore ER homeostasis (132, 133). Research shows that (134, 135), The heavy chain binding protein (BIP), which acts as a molecular chaperone for the ER, is a key regulatory factor in mediating the UPR. BIP recruits misfolded proteins by binding to the ER lumenal domain, causing BIP to dissociate from the conformationally bound states of PERK, IRE1, and ATF6. This inactivates specific transmembrane receptor proteins involved in initiating downstream signaling of the UPR, thereby disrupting protein folding homeostasis. Overexpression of BIP in the ER reduces the activity of IRE1, activating ERS by inhibiting the UPR signaling pathway. Conversely, inhibition of BIP concentration activates UPR by promoting the activity of IRE1, thereby inhibiting ERS (136, 137). Research shows that (138–141), Downregulating the expression of ER molecular chaperones in the peripheral nervous system and CNS can effectively relieve NP by inhibiting ERS. Activating ER molecular chaperones can aggravate NP through the ERS pathway. In SNL model rats, upregulating the expression of ER molecular chaperone BIP in neurons in the dorsal horn of the spinal cord can inhibit UPR by reducing the activity of IRE, activate ERS and thus aggravate NP (142).

### 2.7 Inhibition of glucose metabolism

Glucose is the main source of energy for the brain. Although the mammalian brain only accounts for 2% of body weight, it consumes up to 20% of glucose to maintain normal physiological functions (143, 144). Glucose metabolism is essential for maintaining the physiological functions of the brain. Glucose metabolism mediates neuronal signal transduction processes such as the generation of action potentials and synaptic transmission through the production of ATP and neurotransmitters. When the brain is insufficiently supplied with glucose, it can trigger disturbances in brain glucose metabolism, which seriously affects neuronal signal transmission (145, 146). Glucose needs to enter the brain in synergy with the glucose transporter (GLUTs) family encoded by the SLC2A gene to provide a continuous source of energy for neurons and glial cells in the brain and maintain the normal physiological functions of the brain (147, 148). In addition, glucose transporter 3 (GLUT3) has a strong affinity and transport capacity for glucose uptake, and can also quickly uptake glucose from extracellular fluid with low sugar concentrations to act on neurons, causing neurons to rapidly become excited (149, 150). When NP occurs, glucose uptake rates in numerous brain regions, such as the medial prefrontal cortex (mPFC), somatosensory cortex, dorsolateral thalamus, ACC, and hippocampus, are significantly increased, and NP can be effectively alleviated by reducing glucose metabolism in brain regions related to glucose uptake rates (151–153). High expression of GLUT3 also exacerbates NP by increasing neuronal excitability.

## 3 The analgesic mechanism of EA on NP

**Figure 2:**
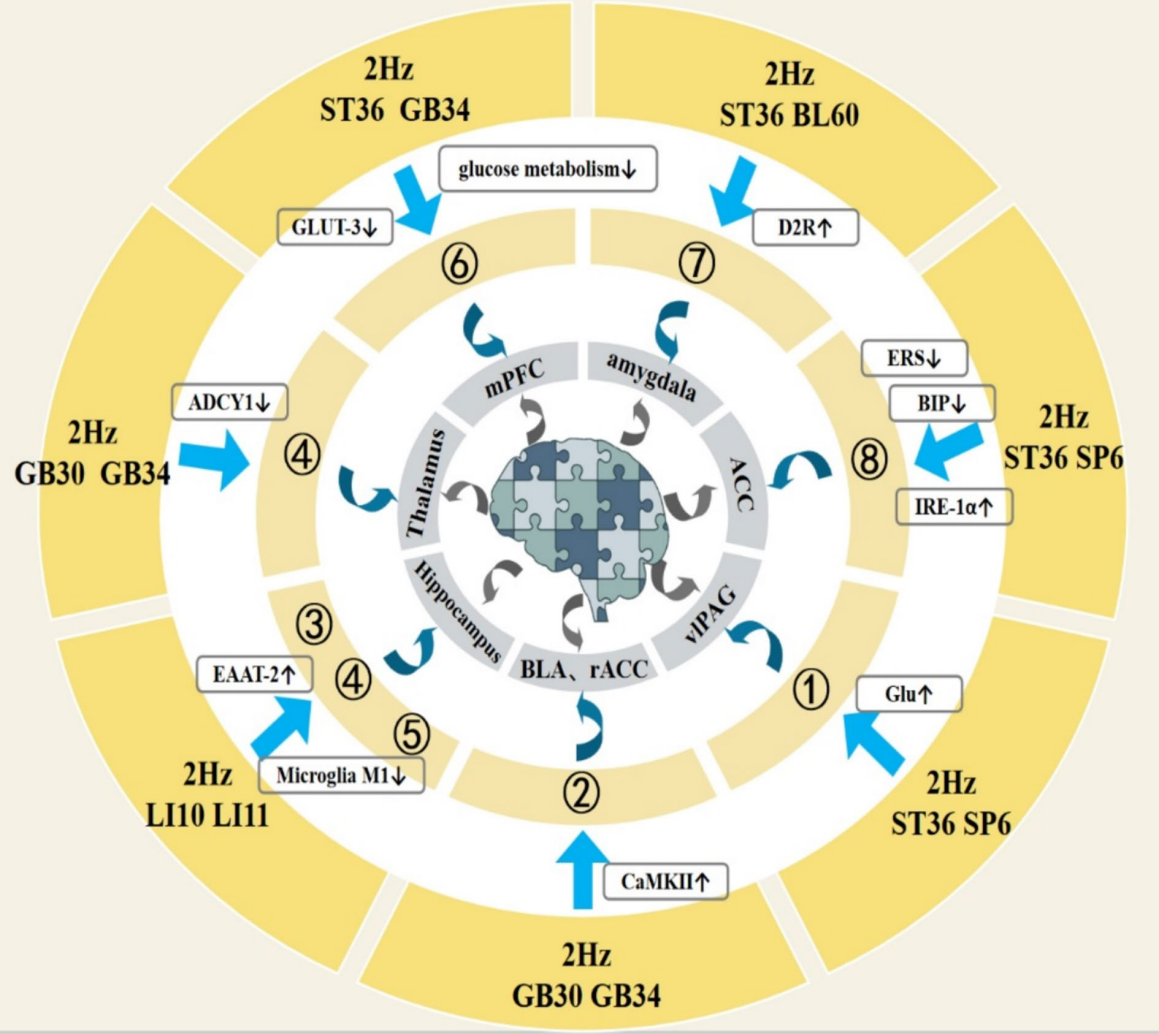
①: Upregulate the content of Glu; ②: Activate CaMKII activity; ③: Upregulates EAAT-2 expression; ④: Inhibits iGluRs expression; ⑤: Inhibit activation of microglia; ⑥: Inhibit glucose metabolism; ⑦: Upregulate D2R expression; ⑧: Inhibit ERS.

**Figure 3:**
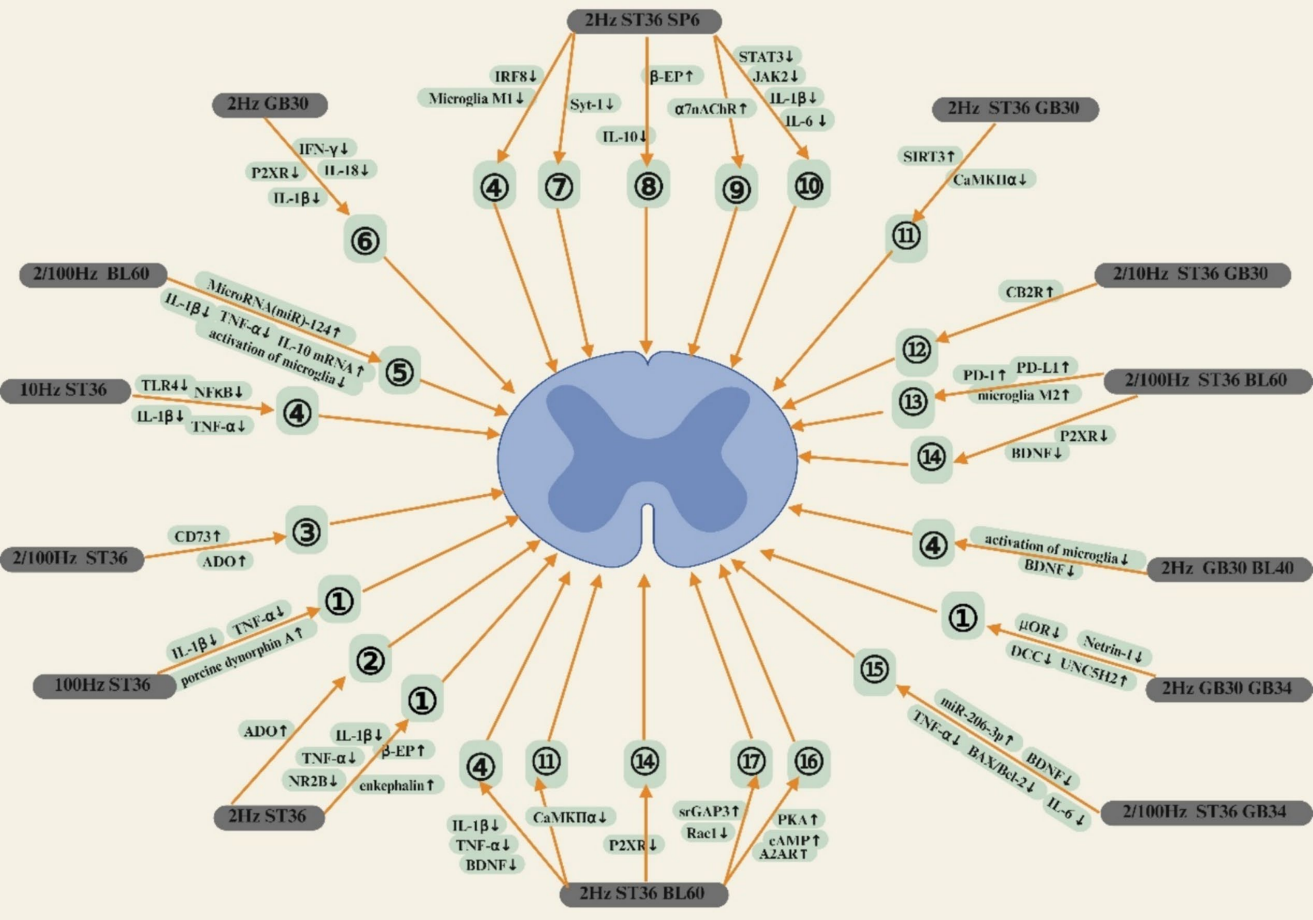
①: Regulates the expression of EOP and its receptors; ②: Expressions that raise ADO; ③: Accelerates the hydrolysis of CD73 and promotes the generation of ADO; ④: Inhibit activation of microglia; ⑤: Promotes the expression of miRNAs, Inhibit activation of microglia; ⑥: Down regulating P2XR expression in microglia; ⑦: Downregulates the expression of Syt-1 and inhibits the release of Glu; ⑧: Inhibit the release of pro-inflammatory factors by microglia; ⑨: Upregulates the expression of the α7nAChR; ⑩: Inhibit the release of pro-inflammatory factors by microglia; ⑪: Inhibits the phosphorylation of CaMKIIα; ⑫: Upregulate the expression of CBR; ⑬: Promote the activation of microglia M2; ⑭: Down-regulating P2XR expression in microglia; ⑮: Promotes the expression of miRNAs; ⑯: Upregulate the expression of A2AR; ⑰: Inhibit dendritic spine remodeling.

EA can relieve NP by regulating the expression of EOP, ADO and their receptors, as well as the content of Glu, and regulating the release of CBR, DR, and α7nAChR. At the same time, EA can also relieve NP by regulating the release of pro-inflammatory factors by microglia and the expression of P2XR. In addition, EA can effectively relieve NP by inhibiting CaMKIIα phosphorylation, dendritic spine remodeling, ERS, and glucose metabolism. The central mechanism of EA in the treatment of NP is shown in Table 1. As illustrated in Figure 2 (EA treatment of NP brain area mechanism) and Figure 3 (EA treatment of NP dorsal horn mechanism).

**Table 1:** Central mechanism of electroacupuncture in the treatment of NP.

	Acupoint	Frequency	Experiment model	Site of action	Mechanism of action	Effect	References
Brain regions	ST36 SP6	2 Hz	SNI	vlPAG	Glu↑	Upregulate the content of Glu	(56)
	ST36 SP6	2 Hz	SNI	BLA, rACC	CaMKII↑	Activate CaMKII activity	(154)	Spinal cord
	GB30 GB34	2 Hz	SNL	Hippocampus	EAAT-2↑, Microglia M1↓	Upregulates EAAT-2 expression and inhibits iGluRs expression; Inhibit activation of microglia	(155)
	LI10 LI11	2 Hz	TP	Thalamus	ADCY1↓	Inhibit the expression of iGluRs	(156)
	ST36 GB34	2 Hz	CCI	Amygdala	D2R↑	Upregulate D2R expression	(157)
	ST36 BL60	2 Hz	CCI	ACC	BIP↓, ERS↓, IRE-1α↑	Inhibit ERS	(158)
	GB30 GB34	2 Hz	CCI	mPFC	glucose metabolism↓, GLUT-3↓	Inhibit glucose metabolism	(159)	ST36	2 Hz	CCI	Dorsal horn of the spinal cord	IL-1β↓, TNF-α↓, β-EP↑, enkephalin↑	Regulates the expression of EOP and its receptors	(161)		ST36	100 Hz	CCI	Dorsal horn of the spinal cord	IL-1β↓, TNF-α↓, dynorphin↑	Regulates the expression of EOP and its receptors	(161)		ST36	2 Hz	PTX	Dorsal horn of the spinal cord	Opioid receptors↑, NR2B↓	Regulates the expression of EOP and its receptors	(162)		ST36	2 Hz	SNI	Dorsal horn of the spinal cord	ADO↑	Expressions that raise ADO	(43)
	ST36	2/100 Hz	CCI	Dorsal horn of the spinal cord	CD73↑, ADO↑	Accelerates the hydrolysis of CD73 and promotes the generation of ADO	(163)
	ST36	10 Hz	PTX	Dorsal horn of the spinal cord	TLR4↓, NFκB↓, IL-1β↓, TNF-α↓	Inhibit activation of microglia	(164)
	BL60	2/100 Hz	CINP	Dorsal horn of the spinal cord	MicroRNA (miR)-124↑, IL-10 mRNA↑, activation of microglia↓, IL-1β↓, TNF-α↓	Promotes the expression of miRNAs, Inhibit activation of microglia	(165)
	ST36 BL60	2 Hz	SNL	Dorsal horn of the spinal cord	A2AR↑, cAMP↑, PKA↑, srGAP3↑, Rac1↓	Upregulate the expression of A2AR; inhibit dendritic spine remodeling	(166, 167)
	ST36 BL60	2 Hz	DNP	Dorsal horn of the spinal cord	P2X4R↓, BDNF↓, IL-1β↓, TNF-α↓, phosphorylation of CaMKIIα↓	Inhibit activation of microglia; Down-regulating P2XR expression in microglia; Inhibits the phosphorylation of CaMKIIα	(96, 168)
	ST36 BL60	2/100 Hz	SNL	Dorsal horn of the spinal cord	PD-L1↑, PD-1↑, activation of microglia M2↑, MAPK↓, activation of microglia↓, P2X4R↓, BDNF↓	Promote the activation of microglia M2; Inhibit activation of microglia;Down-regulating P2XR expression in microglia	(91, 169)
	ST36 SP6	2 Hz	SNI	Dorsal horn of the spinal cord	Syt-1↓, Microglia M1↓, IRF8↓	Downregulates the expression of Syt-1 and inhibits the release of Glu; Inhibit activation of M1 microglia	(170, 171)
	ST36 SP6	2 Hz	SNI	Dorsal horn of the spinal cord	α7nAChR↑, IL-1β↓, STAT3↓, JAK2↓, IL-6 mRNA↓	Upregulates the expression of the α7nAChR; Downregulate the expression of inflammatory cytokines	(78, 172)
	ST36 SP6	2 Hz	SNL	Dorsal horn of the spinal cord	IL-10↑, β-EP↑	Inhibit the release of pro-inflammatory factors by microglia	(173)
	GB30	2 Hz	CCI	Dorsal horn of the spinal cord	IFN-γ↓, P2X4R↓, P2X7R↓, IL-1β↓, IL-18↓	Down-regulating P2XR expression in microglia	(98, 174)
	ST36 GB30	2 Hz	CCI	Dorsal horn of the spinal cord	SIRT3↑, phosphorylation of CaMKIIα↓	Inhibits the phosphorylation of CaMKIIα	(107)
	ST36 GB30	2/10 Hz	CCI	Dorsal horn of the spinal cord	CB2R↑	Upregulate the expression of CBR	(175)
	GB30 BL40	2 Hz	SNI	Dorsal horn of the spinal cord	activation of microglia↓, BDNF↓	Inhibit activation of microglia	(176)
	GB30 GB34	2 Hz	PHN	Dorsal horn of the spinal cord	μOR↓, Netrin-1↓, DCC↓, UNC5H2↑	Regulates the expression of EOP and its receptors	(177)
	ST36 GB34	2/100 Hz	CCI	Dorsal horn of the spinal cord	miR-206-3p↑, BDNF↓, BAX/Bcl-2↓, IL-6↓, TNF-α↓	Promotes the expression of miRNAs	(178)

↑Indicates an increase or upward adjustment; ↓indicates a decrease or downward adjustment.

### 3.1 The brain mechanism of EA in the treatment of NP

2 Hz EA ZuSanLi (ST36) and SanYinJiao (SP6) can effectively relieve NP by increasing the content of Glu in the vlPAG of SNI model mice and activating the activity of CaMKII neurons between the lateral basolateral amygdala and the anterior cingulate cortex of SNI model rats (56, 154).

2 Hz EA HuanTiao (GB30) and YangLingQuan (GB34) relieve NP (155).

2 Hz EA QuChi (LI11) and ShouSanLi (LI10) significantly down-regulate the expression of ADCY1, a downstream protein of NMDAR in the rat thalamus, alleviating NP (156).

2 Hz EA ST36, GB34 can relieve NP by increasing D2R expression in the amygdala of CCI rats (157).

2 Hz EA ST36 and KunLun (BL60) promote IRE1 activity and UPR signaling by inhibiting the expression of the ER molecular chaperone BIP in the ACC brain region of CCI model rats, and inhibit ERS in the nervous system to relieve NP (158).

2 Hz EA GB30, GB34 alleviates NP by reducing glucose metabolism and GLUT-3 content in the mPFC of CCI model rats (159).

### 3.2 The dorsal horn of the spinal cord mechanism of EA in the treatment of NP

Research shows that (160), 2 Hz EA can accelerate the release of *β*-EP and enkephalin in the CNS and produce an analgesic effect on NP by binding to μOR and δOR, while 100 Hz EA produces an analgesic effect by increasing the release of enkephalin and further activating κOR. Both 2 Hz and 100 Hz EA ST36 can reduce the release of inflammatory cytokines such as IL-1β and TNF-*α* and other inflammatory cytokine release. Among them, 2 Hz EA ST36 can relieve NP by promoting the release of β-EP and enkephalin, while 100 Hz EA ST36 has an analgesic effect on NP by promoting the secretion of dynorphin (161). In addition, the 2 Hz EA ST36 can effectively relieve NP by upregulating the expression of opioid receptors in the dorsal horn of the spinal cord and inhibiting the phosphorylation of the NR2B subunit of NMDA receptors in mice with a PTX-induced NP model (162). 2 Hz EA ST36 can also relieve NP by increasing the expression level of ADO in the dorsal horn of the spinal cord of SNI model mice (43). 2/100 Hz EA ST36 can relieve NP by upregulating the expression level of ADO by accelerating the hydrolysis of CD73 in the dorsal horn of the CCI model rat spinal cord (163). 10 Hz EA ST36 also has an analgesic effect on NP by inhibiting PTX-induced activation of microglia in the dorsal horn of the spinal cord and production of pro-inflammatory cytokines in NP model rats (164).

2/100 Hz EA BL60 can inhibit microglial activation and alleviate NP by increasing the expression level of miR-124 in CINP model rats (165). 2 Hz EA ST36 and BL60 can effectively relieve NP by inhibiting dendritic spine remodeling by activating the A2AR/cAMP/PKA signaling pathway in the dorsal horn of the SNL rat model and regulating the srGAP3/Rac1 signaling pathway (166, 167). In addition, the 2 Hz EA ST60 and BL60 can also relieve NP by inhibiting the expression level of P2X4R and the phosphorylation of CaMKIIα in microglia activated in the dorsal horn of the spinal cord of DNP model rats (96, 168). 2/100 Hz EA ST36, BL60 can effectively relieve NP by increasing the expression levels of PD-L1 and PD-1 in the dorsal horn of the SNL model rat spinal cord, promoting the polarization of M2 microglia and inhibiting the MAPK signaling pathway and the expression level of P2X4R (91, 169).

2 Hz EA ST36 and SP6 can reduce the production of pro-inflammatory factors and thus relieve NP by down-regulating the expression of Syt-1 in neurons and glial cells in the dorsal horn of the rat spinal cord and inhibiting the release of Glu and the activation of microglia type M1 in the SNI model (170, 171). In addition, the 2 Hz EA ST36 and SP6 not only upregulate the expression of α7nAChR in the dorsal horn of the spinal cord of SNI model rats, but also reduce the release of inflammatory cytokines and inhibit the JAK2/STAT3 signaling pathway to relieve NP (78, 172). The IL-10/*β*-EP pathway can also relieve NP by upregulating the expression of IL-10 and β-EP in microglia in the dorsal horn of the spinal cord of rats in the SNL model (173).

2 Hz EA GB30 can relieve NP by inhibiting the excessive release of IFN-*γ*, IL-1β, and IL-18 in the dorsal horn of the CCI model rat spinal cord, thereby inhibiting the expression of P2X4R and P2X7R in microglia (98, 174). 2 Hz EA ST36, GB30 inhibits CaMKIIα phosphorylation and relieves NP by upregulating SIRT3 expression in the dorsal horn of the rat spinal cord in the CCI model (107). 2/10 Hz EA ST36, GB30 can relieve NP by upregulating the expression level of CB2R in the dorsal horn of the rat spinal cord in the CCI model (175). In addition, 2 Hz EA GB30 and WeiZhong (BL40) can reduce BDNF expression in the dorsal horn of the spinal cord of SNI model rats, improve the anti-inflammatory function of microglia, and relieve NP (176). 2 Hz EA GB30, GB34 can relieve NP by activating the release of μOR in the dorsal horn of the PHN model rat, reducing the expression levels of Netrin 1 (NTN1), and its receptor DCC in neurons in the dorsal horn, and increasing the content of UNC5H2, the receptor for NTN1 (177). 2/100 Hz EA ST36, GB34 can relieve NP by increasing the expression of miR-206-3p in the dorsal horn of the CCI rat model, inhibiting the expression level of BDNF and the content of pro-inflammatory cytokines BAX/Bcl-2, TNF-*α*, and IL-6 (178).

## 4 Discussion

In recent years, the central analgesic mechanisms of EA on NP have mainly included: regulating the release of pain-related neurotransmitters and receptors, up-regulating the expression of miRNAs, inhibiting the activation of microglia, CaMKIIα phosphorylation, dendritic spine remodeling, ERS, glucose metabolism, etc. With the deepening of EA research on the analgesic mechanism of NP, more research results have been obtained. Compared with traditional drug therapy, EA has the advantages of excellent efficacy, few side effects and low cost, which provides EA treatment of NP with irreplaceable prerequisites. However, current research on NP focuses more on the mechanism, and the experimental model used also centers on rats, lacking clinical trials and relevant biochemical indicators using human subjects as the research object. This has directly led to the clinical efficacy of EA in the treatment of NP being questioned and controversial. Therefore, future research should focus more on randomized controlled trials to confirm the clinical efficacy of electroacupuncture in the treatment of NP, so that EA can go global and become an internationally recognized means and solution for the treatment of NP.

The author found that after collating and summarizing the analgesic mechanism of EA at the central level for NP over the past 10 years, EA has a good analgesic effect on various animal models of NP. EA is mostly used to treat NP by acupuncture at lower limb acupoints such as ST36, BL60, and SP6 at 2 Hz. As NP is a type of pain caused by various diseases, it not only leads to numerous mechanisms of NP, but also to diverse animal models. This has led to the need for continued clarification and in-depth research on EA for the treatment of NP. Second, the research on the mechanism of EA analgesia for NP at the central level involving the relevant brain areas is far less than that at the spinal cord level. In the future, the mechanism of EA in the treatment of NP at the central brain area should be improved, and the pain mechanisms between brain areas and between brain areas and the dorsal horn of the spinal cord should be studied by making full use of neuron tracing and multi-omics technology.

This article reviews the central mechanism of EA in the treatment of NP. It is believed that with the development and progress of emerging technologies such as artificial intelligence, the mystery of EA’s treatment of NP through a holistic, multidimensional, multi-level, and multi-faceted regulatory effect will finally be completely revealed. At the same time, human beings’ means of treating NP will also become increasingly diverse and abundant. It is believed that in the near future, NP, with its complex mechanism, will definitely be conquered by human beings.

## Glossary

ADO: Adenosine
ARC: Arcuate nucleus of the hypothalamus
ATP: Adenosine triphosphate
ADP: Adenosine diphosphate
AMP: Adenosine monophosphate
A2ARs: Adenosine A2A receptor
A3Rs: Adenosine A3 receptor
ADCY: Adenylate cyclase
ACC: Anterior cingulate cortex
ACh: Acetylcholine
ATF-6: Activating Transcription Factor 6
BDNF: Brain-derived neurotrophic factor
BIP: Heavy chain binding protein
CCI: Chronic compression of the sciatic nerve
CCI-ION: Trigeminal neuralgia
CBR: Cannabinoid receptor
CD39: Extracellular nucleoside triphosphate hydrolase
CD73: Extracellular-5′-nucleotidase
cAMP: cyclic adenosine monophosphate
CINP: Cisplatin-induced neuropathic pain
CB2R: Cannabinoid receptor 2
CB1R: Cannabinoid receptor 1
CaMKII: Ca^2+^/calmodulin kinase II
CNS: Central nervous system
DA: Dopamine
DR: Dopamine receptor
D1R: Dopamine type 1 receptor
D2R: Dopamine type 2 receptor
DNP: Diabetic neuropathic pain
EA: Electroacupuncture
EOP: Endogenous opioid peptide
EAAT-2: Glutamate Transporter 2
ECS: Endocannabinoid system
ER: Endoplasmic reticulum
ERS: Endoplasmic reticulum stress
EGR1: Early growth response gene 1
Glu: Glutamic acid
GLUTs: Glucose transporter
GLUT3: Glucose transporter 3
iGluRs: ionotropic glutamate receptor
IFN-γ: Interferon-γ
IRF8: Interferon regulatory factor 8
IRE1: Inositol requires enzyme 1
JAK-2: Janus-kinase-2
LPS: Lipopolysaccharide
miRNA: microRNA
mPFC: medial prefrontal cortex
NP: Neuropathic Pain
NMDA: N-methyl-D-aspartic acid
NMDAR: N-methyl-D-aspartate receptor
nAChR: Nicotinic acetylcholine receptor
NF-κB: Nuclear factor kappa-B
ncRNA: Non-coding RNA
NTN1: Netrin 1
PHN: Post-herpetic neuralgia
PKA: Protein kinase A
PKC: Protein kinase C
PTX: Paclitaxel
P2XR: Purinergic receptor
PD-L1: Programmed death ligand 1
PD-1: Programmed death receptor 1
PERK: Protein kinase R-like endoplasmic reticulum kinase
ROS: Reactive oxygen species
Rac1: Ras-related C3 botulinum toxin substrate 1
SNL: Spinal nerve ligation
SNI: Selective nerve injury
SAH: Adenosine-L-homocysteine
Syt-1: Synaptotagmin-1
STAT3: Transcription activator-3
STZ: Streptozotocin
SIRT3: Sirtuin 3
SRGAP3: SLIT-ROBO Rho GTPase Activating Protein 3
TGF-β: Transforming growth factor-β
TNF-α: Umor necrosis factor-alpha
TLR4: Toll-like receptor 4
UPR: Unfolded protein response
vlPAG: ventrolateral periaqueductal gray
α7nAChR: α7 nicotinic acetylcholine receptor
β-EP: beta-endorphin
μOR: μ-opioid receptor
δOR: δ-opioid receptor
κOR: κ-opioid receptor
